# Development of burnout over time and the causal order of the three dimensions of burnout among male and female GPs. A three-wave panel study

**DOI:** 10.1186/1471-2458-11-240

**Published:** 2011-04-18

**Authors:** Inge Houkes, Yvonne Winants, Mascha Twellaar, Petra Verdonk

**Affiliations:** 1 Maastricht University, Researchschool CAPHRI, Department of Social Medicine, PO Box 616, 6200 MD Maastricht, The Netherlands; 2 Maastricht University, Researchschool CAPHRI, Department of General Practice, PO Box 616, 6200 MD Maastricht, The Netherlands; 3 Maastricht University, Researchschool CAPHRI, Department of Genetics and Cell Biology, PO Box 616, 6200 MD Maastricht, The Netherlands

## Abstract

**Background:**

A good understanding of the aetiology and development of burnout facilitates its early recognition, prevention and treatment. Since the prevalence and onset of this health problem is thought to differ between men and women, sex must be taken into account. This study aims to assess the prevalence and development of burnout among General Practitioners (GPs). In this population the prevalence of burnout is high.

**Methods:**

We performed a three-wave longitudinal study (2002, 2004, 2006) in a random sample of Dutch GPs. Data were collected by means of self-report questionnaires including the Maslach Burnout Inventory. Our final sample consisted of 212 GPs of which 128 were male. Data were analyzed by means of SPSS and LISREL.

**Results:**

Results indicate that about 20% of the GPs is clinically burned out (but still working). For both sexes, burnout decreased after the first wave, but increased again after the second wave. The prevalence of depersonalization is higher among men. With regard to the process of burnout we found that for men burnout is triggered by depersonalization and by emotional exhaustion for women.

**Conclusions:**

As regards the developmental process of burnout, we found evidence for the fact that the aetiological process of burnout, that is the causal order of the three burnout dimensions, differs between men and women. These sex differences should be taken into account in vocational training and policy development, especially since general practice is feminizing rapidly.

## Background

There is an ongoing debate about the question whether, in general, working women can be expected to be more at risk for burnout than men [1,2]. We do know that Dutch *women *have a higher risk of getting disabled for work due to psychological complaints [3] and that Dutch highly educated women report a higher prevalence of work-related fatigue than men [4]. Next to this, there is still a considerable lack of clarity about the causal order of the three burnout dimensions emotional exhaustion (EE; the depletion of emotional resources), depersonalization (DP; the development of a negative, callous and cynical attitude towards the recipients of one's services) and reduced personal accomplishment (PA; the tendency to evaluate one's work negatively, feelings of insufficiency and poor professional self esteem). A good understanding of the aetiology and development of burnout though, could facilitate the early recognition and treatment of burnout [5-7]. Most researchers agree, that burnout develops gradually over time, and can be considered a process [7]. Considering the above, it is also relevant to consider sex differences in the development of burnout as the onset of this syndrome might be different for men and women, and hence, the early burnout signals might differ for men and women as well [6]. The present study aims to address these issues by means of a longitudinal three-wave study among a sample of the understudied population of General Practitioners.

### Burnout among General Practitioners

Among General Practitioners (GPs) the prevalence of burnout and other stress related complaints has increased during the last decade [8,9]. The organization of the work of GPs as well as their working conditions (i.e., a high workload, organizational problems) may play a role in the high burnout prevalence among doctors. Several studies among GPs show that work factors such as time pressure, emotional demands, patient factors (e.g., dealing with problem patients), and night calls are important problems associated with ongoing stress in the working lives of GPs [9-12]. Additionally, we know that the conditions of medical work in primary care suggest that physicians are particularly prone to work-family interference [13] and that work-family conflict is an important cause of burnout as well [14]. All in all, it is not surprising that burnout levels among GPs are high. A recent Dutch study [15] indicates that GPs score higher on burnout than the general working population in the Netherlands. A recent literature review [16] with regard to burnout among medical residents (including family practitioners) revealed that the prevalence of burnout among medical residents ranged from 18 up to 82%.

### Sex differences in burnout

The percentage of female GPs in The Netherlands has been increasing up to 45% in 2010 [17,18]. Considering this gender shift that is taking place in a profession that has historically been dominated by men, sex differences need to be included in burnout research among GPs. Various reasons exist for including sex in occupational health research: *exposure *to occupational risks differs for men and women - even within the same jobs -, men and women may *experience *the same jobsite differently, and may differ in their *responses *to occupational exposure [19]. In addition, it is argued that sex discrimination in the workplace is a source of stress and can cause a lack of social support for women, which is also a risk factor for burnout. Moreover, women are found to have less effective professional networks, are confronted with the negative consequences of stereotyping, tokenism, and a high workload due to unequal division of household and caring tasks [20].

Empirical results with regard to the prevalence of burnout among men and women are inconsistent. When studies do report sex differences it is often difficult to interpret these differences because of an unequal distribution of men and women, or the confounding effect of occupation and/or marital status, implying that sometimes sex differences in fact reflect occupational differences [21]. This makes it also difficult to compare burnout prevalences *between *studies. Table 1 provides an overview of burnout studies that report about burnout prevalence among men and women. It can be concluded from this Table that women - in general - score higher on emotional exhaustion, while men score higher on depersonalisation and personal accomplishment.

**Table 1:** Prevalence of burnout among men and women

*Reference*	*Sample(s)*	*Significant gender differences in burnout prevalence*
[1]	Literature review	Females score higher on EE Males score higher on DP
[2]	694 female and 2225 male Dutch employees	Females score higher on EE Males score higher on PA
[7]	Literature review	Females score higher on EE Males score higher on DP
[21]	Literature review	Females score higher on EE Males score higher on DP Males score higher on PA
[49]	277 female and 216 male Greek teachers	Females score higher on EE
[50]	317 female and 77 male Dutch nurses	Males score higher on EE ^a^ DP and PA not measured
[51]	93 female and 138 male athletics coaches	Females score higher on EE Males score higher on PA
[52]	218 Chinese **doctors **and nurses	Females score higher on EE
[53]	403 female and 664 male Dutch academia	No differences on EE DP and PA not measured
[54]	227 female and 243 male Canadian teachers	Males score higher on DP
[55]	552 female and 217 male Greek Cypriot school teachers	Females score higher on EE
[56]	122 female and 141 male American faculty members	Females score higher on EE Males score higher on DP
[57]	32 female and 68 male American **family practice residents**	Males score higher on DP Among males DP increased over time
[58]	163 female and 239 male South African **doctors**	Females score higher on EE Males score higher on PA
[59]	347 female and 248 male American clinical psychologists	Males score higher on DP
[60]	154 female and 141 male Turkish teachers	Females score higher on EE Males score higher on DP
[61]	1677 female and 1634 male **Finnish physicians**	Females score higher on EE Males score higher on DP
[62]	336 female teachers and 219 male Spanish teachers	Females score higher on EE Males score higher on DP

^a ^These findings are explained by the number of working hours. Men work more hours, and this contributes significantly to emotional exhaustion. In addition, the numerical domination of women in nursing might be a protective factor against burnout for women in this sector.

### The development of burnout: causal order of the three burnout dimensions

**Figure 1:**
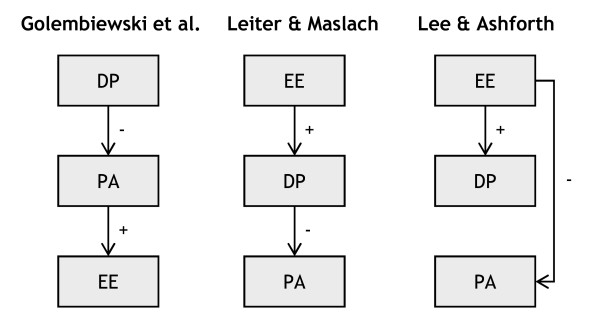
**Overview of three theoretical burnout models**.

One issue that has continued to interest burnout researchers and practitioners concerns the development of burnout: the relationships among the three burnout dimensions. It is generally assumed that the three burnout dimensions do not develop simultaneously, and hence, knowledge about the causal order of the three burnout dimensions can be relevant for the early recognition of burnout and the identification of "high-risk" people who could be targeted for early, preventive interventions [6,7]. Several researchers have interpreted the associations among the three dimensions as resulting from an underlying causal process that reflects the development of burnout. Theoretically, three prominent models exist that describe this process: the phase model of Golembiewski, Munzenrider and Stevenson [22], Leiter and Maslach's process model [23], and Lee and Ashforth's [24] model [25]. Golembiewski et al. argue that in the process of burning out depersonalization is experienced first, since some level of detachment is necessary in service settings. At a certain point, detachment becomes depersonalization, undermining performance and triggering emotional strain. In contrast with Golembiewski and his colleagues, Leiter and Maslach argue that emotional exhaustion is the first burnout dimension to develop. They consider burnout as a response to chronic job stress. High job demands trigger emotional exhaustion, which in turn leads to depersonalization as an attempt to cope with the stressors. When these feelings of depersonalization persist, the achievement of work goals can be hampered and feelings of reduced personal accomplishment may develop [23]. Lee and Ashforth finally, propose a variation on the latter two models, based on their attempt to compare these models. Just like Leiter and Maslach, they consider depersonalization to develop from emotional exhaustion. However, they additionally propose that reduced personal accomplishment develops independently from depersonalization. In fact, they state that reduced personal accomplishment is evoked directly by emotional exhaustion [24]. See Figure 1 for an overview of the models.

In their 2005 publication, Taris et al. [25] report about their systematic literature search in PsycINFO and Medline (updated to May 2005) with regard to the causal relationships among the three burnout components. They only found seven studies that met their inclusion criteria (i.e., a longitudinal design, including at least two MBI components, and including a causal interpretation of the relationships among the burnout dimensions). They concluded that longitudinal evidence for any causal ordering of burnout dimensions is virtually absent. Most longitudinal associations reported in these studies refer to stability effects (e.g., EE at T1 is a good predictor of EE at T2). Therefore, Taris and colleagues performed two longitudinal studies themselves (among oncology care providers and teachers) in order to find evidence for a causal order of burnout dimensions. They tested the three theoretical models mentioned above, as well as a model of their own, the latter being an integration of the Leiter and Maslach, and Lee and Ashforth models, which additionally includes feedback effects of depersonalization on both emotional exhaustion and personal accomplishment. These feedback effects were included to test whether depersonalization serves as a coping strategy, as is argued by many other authors [23,24]. In this light it is remarkable that in none of the existing theoretical models depersonalization is explicitly treated as a coping strategy and as a result, it has hardly been tested as such. Taris and colleagues found that both the Leiter and Maslach and the Lee and Ashforth models fitted the data better than the Golembiewski model. Their own model fitted the data best, although the direct effect of emotional exhaustion on personal accomplishment was not significant in both samples. They concluded that high levels of emotional exhaustion trigger depersonalization, and that in turn high levels of depersonalization reduce feelings of personal accomplishment. Emotional exhaustion and personal accomplishment appeared not to be related. In the teacher sample they also found a feedback effect from depersonalization on emotional exhaustion, implying that depersonalization is a dysfunctional coping strategy. They did not distinguish between men and women in their study despite the unequal numbers of men and women in their sample and the fact that both samples were taken from highly sex segregated professions.

### Sex differences in the development of burnout

We hypothesize that the inconsistent findings of the effect of depersonalization as a coping strategy in Taris' study is a result of the fact that sex was not taken into account in their analyses [26]. The effect or working mechanism of depersonalization may differ between men and women because of differences in gender socialisation. Gender socialisation theory poses that among boys and men, assertiveness and independence is stimulated, just as repelling feelings of vulnerability. They are taught to be aggressive, non-emotional and goal-oriented, and not to show weakness. This socialisation may lead to the incapability of men to solve internal conflicts and to their repression of internal conflicts, which may in turn lead to higher chances of depersonalization [27,28]. For women, it is stated that gender socialisation leads to attitudes of tenderness, sharing, being considerate and kind, and to repelling aggressive behaviour. Thus, in similar circumstances men may report higher levels of depersonalization. In addition, for men depersonalization may be the core component which triggers off the whole burnout process, while for women emotional exhaustion may be the triggering factor. In line with this suggestion, Taris and colleagues did find a feedback effect from depersonalization on emotional exhaustion in the sample with the most male participants (i.e., the teachers).

With regard to coping, it has been shown that women report using all coping behaviours more often than men, and in addition, have a larger coping repertoire (i.e., they use a broader variety of coping styles) [29]. With regard to preferences for particular coping strategies, research is rather ambiguous, although women tend to favour emotion-focused coping, while men have a preference for both problem-focused coping and avoidance. Eaton and Bradley [29] explain these sex differences in coping by the differences in stress appraisal between men and women (that is, women tend to appraise the same stressors as more severe than men), and by gender socialization processes. Depersonalization is sometimes considered as a - dysfunctional - coping strategy [7] which can be considered similar to avoidance. More than women, men may use depersonalization as a way to deal with the emotional demands inflicted on them at work, while women tend to use emotion-focused coping, wear themselves out, and become emotionally exhausted. Only then, in a later stage than men, they develop the depersonalising attitude towards their patients.

### Aim of the study

Besides the study of Taris and his colleagues, there is hardly any evidence for the causality of the relationships among the three burnout dimensions. Furthermore, the few high quality longitudinal studies that do exist regarding the burnout process did not take sex into account. Based on the theoretical underpinnings above, we formulated the following research questions:

1. What is the prevalence of burnout among male and female GPs and how does this change over time?

2. What is the causal order of the three dimensions of the burnout syndrome among male and female GPs?

Prior to our field study, we updated the search of Taris and colleagues [25] to July 2010. We searched the PsycINFO and Medline databases using the same key words (i.e., "burnout", "longitudinal", "panel", "development", "causal", "Maslach Burnout Inventory", "MBI", and "phase model" in various combinations) and inclusion criteria as Taris and colleagues. We only found two additional studies that met the inclusion criteria [30,31]. The first study [30] was performed among 79 beginning Australian teachers (84% females). The study had a 4-wave complete panel design and covered a two-year period. Hierarchical regression analyses were used to analyse that data. No lagged effects were reported, only stability effects. The second study [31] was performed among 2 two-wave samples (302 staff members of a nursing home, 85% females, and 341 secretaries of a civil service organization, 55% females). In this study, six theoretical process models of burnout were tested, five taken from the literature, and one developed by the researchers. Results showed several lagged effects: EE was related to DP, and PA was predicted by both EE and DP. Sex was not taken into account.

## Methods

### Design, procedure and participants

We conducted a full panel design with three waves (2002, 2004, and 2006) and used self-report questionnaires to measure the study variables. These 2-year time lags seem long enough to measure possible changes in individual scores, and not too long with regard to non-response [32]. The questionnaires were sent out by mail to the working addresses of the GPs. Participants could return the questionnaires by means of a stamped and addressed return envelope. In between the first and second measurement, the GPs received a short letter including feedback regarding their burnout scores. In between the second and third measurement, the GPs received some feedback about their burnout scores as well, albeit somewhat shorter than the first time. All data were treated confidentially.

The study population consisted of 685 GPs working on a permanent basis, randomly selected from the Dutch GP population. All Dutch GPs are registered by NIVEL (the Netherlands institute for health care research). In 2002, 7341 people (5588 men and 1753 women) were working as a GP in the Netherlands [33]. Our 2002 sample was selected using the database of NIVEL. We took a disproportionate sample with respect to sex: 50% of our study population was female, while in the total Dutch GP population in 2002 28% was female. When comparisons between strata are sought, this type of disproportionate sampling is adequate [19,34]. GPs who did not respond within 3 weeks were encouraged by telephone to fill out the questionnaire. By filling out and returning the questionnaire GPs gave their informed consent to participating in the study. The study was not submitted to an ethical committee because according to Dutch law (Wet Medisch-Wetenschappelijk Onderzoek met Mensen/Medical Research Involving Human Subjects Act), surveys only have to submitted to an ethical committee in a limited number of situations which do not apply to the current study.

### Measures

*Burnout *was measured by means of the Dutch version of the Maslach Burnout Inventory (MBI) [35], that measures the three burnout dimensions emotional exhaustion (eight items), depersonalization (five items) and personal accomplishment (seven items). The MBI is a widely used instrument to measure burnout. Two example items of the emotional exhaustion scale are "I feel emotionally drained by my work" and "I feel fatigued when I get up in the morning and have to face another day on the job". Two example items of the depersonalization scale are "I worry that this job is hardening me emotionally" and "I feel I treat some patients as if they were impersonal objects". Two example items of the personal accomplishment scale are "I have accomplished many useful things in my work" and "I deal very effectively with the problems of my patients". Items are scored on a 7-point rating scale with fixed anchors that range from "never" to "every day". Previous studies have indicated that internal consistencies, factorial and construct validity of the MBI are satisfactory and stable [7]. The Cronbach's alphas of the MBI in the current study (T1) were satisfactory as well: .87 for emotional exhaustion, .73 for depersonalization, and .77 for personal accomplishment. Skewness and kurtosis of all three burnout dimensions lie within the acceptable limits (ranging from -1 to 1). The calculation of the percentage of GPs that is clinically burned is based on the - rather conservative - Dutch cut-off points for primary health care workers [35]. A high score on emotional exhaustion, combined with a high score on either depersonalization or personal accomplishment, indicates cases of clinical burnout. Cut-off points for the three burnout dimensions correspond to the 95^th ^percentile for emotional exhaustion, and to the 75^th ^percentile for depersonalization and personal accomplishment. Note that the MBI originally was not developed as an individual diagnostic tool, and that only for the Netherlands clinically valid cut-off points seem to be available [7,35].

### Data analyses

In the first place we performed several preliminary analyses (*M*, *SD*, burnout-prevalence and Pearson correlations for the total group, as well as men and women separately). In order to answer the first research question (change in burnout prevalence over time and sex differences herein) we performed General Linear Model repeated measures (GLM 4).

In order to answer research question 2, regarding the causal development of burnout, we used a cross-lagged panel design (CLPD) and tested a sequence of competing, nested structural equation models in several steps [36-38]. CLPD requires the use of complete data (i.e., panel data), that is data from respondents who participated at all measurement points. We arrived at our final study sample by means of listwise deletion [32]. This procedure is inherent to CLPD. The CLPD design is highly recommended by many researchers in the field of work and organizational psychology particularly when the aim is to find evidence for causation [32,38-40]. The problem of attrition that can hamper this design is usually recognized, but comparing path estimates based on different samples is problematic as well [40]. By means of CLPD it is possible to analyse men and women separately, hence we did not treat sex as a confounder [19]. Controlling for sex while it is not a confounder - which it rarely is - may result in an underestimation of the true exposure-effect relationship. We corrected for stability effects in all models, in order to ascertain that cross-lagged relationships we may find, are not influenced by high stability levels of the burnout dimensions. The final panel group hardly contained missing data, hence ML (maximum Likelihood) estimation was used to estimate the structural equation models [37]. To assess the fit of the various models, several commonly used fit indices were used [37,41]: the chi-square statistic (*Χ^2^*), the root mean square error of approximation (RMSEA), the non-normed fit index (NNFI), the comparative fit index (CFI) and the standardized root mean square residual (SRMR). With regard to specific relationships, LISREL provides *t*-values indicating the significance of the specified relationships, and the so-called "modification-indices". These modification indices provide information as to what specific relationships should be added to the model, when theoretically plausible, in order to improve the fit between the hypothesized model and the data. Finally, competing models were compared by means of the chi-square difference test [37] and the Akaike information criterion (AIC) [41].

We did not specify the measurement models of the three burnout measures, as these measures have generally proven to be valid and reliable in earlier research [35].

## Results

### Non-response and sample characteristics

**Figure 2:**
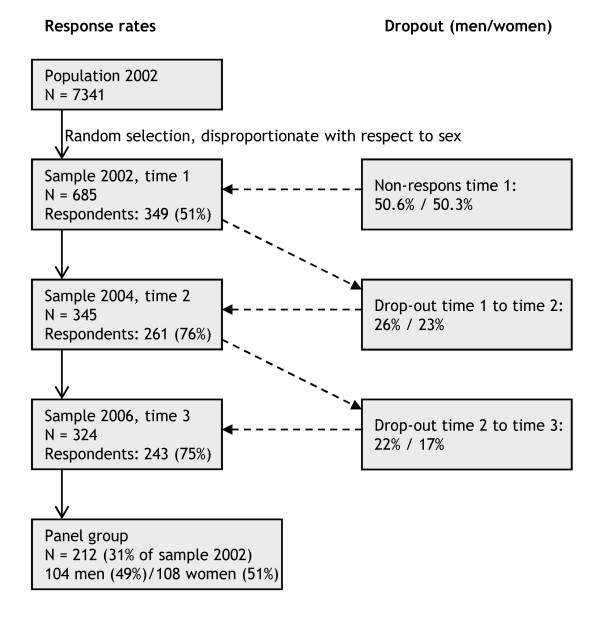
**Response rates at time 1, time 2 and time 3**.

Figure 2 provides an overview of the response rates at the three measurement points and the dropout rates. Only GPs who responded and still practiced received a questionnaire at a next wave. It is a common feature of many panel studies that a large part of the initial sample is lost due to attrition (69% in the current study). This may have implications for external validity of the findings. Furthermore, Figure 2 shows that the dropout among men is slightly higher than among women, mainly due to the fact that particularly male GPs reached the retirement age during our study. In order to rule out selection problems due to panel loss, we determined by means of *t*-tests whether there were mean differences between GPs in the panel group and the dropouts with regard to the three burnout dimensions. There were no significant mean differences at both time 1 and time 2. When we consider these mean differences for men and women separately, we see that among women, the level of emotional exhaustion at time 1 is somewhat higher among the dropouts than in the panel group, although the mean difference is less than one half of the standard deviation. We did not find any differences on time 2. For men, there were no mean differences on time 1. On time 2, we found that the dropouts scored somewhat lower on depersonalization than the panel group, again the mean difference was less than one half of the standard deviation. In sum, the non-response analyses showed that there were hardly any differences regarding the burnout dimensions and only slight differences regarding sex. This type of missing data is called stratified MCAR (Missing Completely at Random) and justifies using listwise deletion as method to handle missing data when sex is included in the analyses, as is the case in this study [42].

The final panel group comprised 104 (49%) men and 108 (51%) women. They reported a mean age of 48.44 years (*SD *= 6.25). The women were significantly younger (mean age = 42.96, *SD *= 5.45). 77% of the respondents at time 1 had children (*M *= 1.7) who were living at home, the mean age of the youngest child was 8.4 years (*SD *= 5.8) among women and 11.9 years (*SD *= 6.2) among men. Finally, at time 1, 81% of the male GPs worked 40 hours or more. For female GPs this percentage was 31%.

### Preliminary analyses and research question 1: change in prevalence of burnout over time among male and female GPs

**Figure 3:**
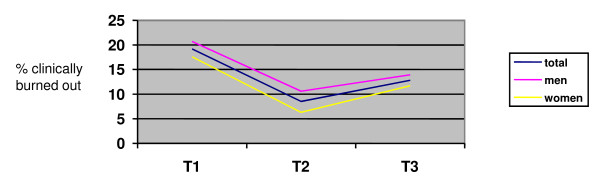
**The development of the percentage of clinically burned out GPs over time**. Norms were deducted from the UBOS manual of Schaufeli and Van Dierendonck [34].

**Figure 4:**
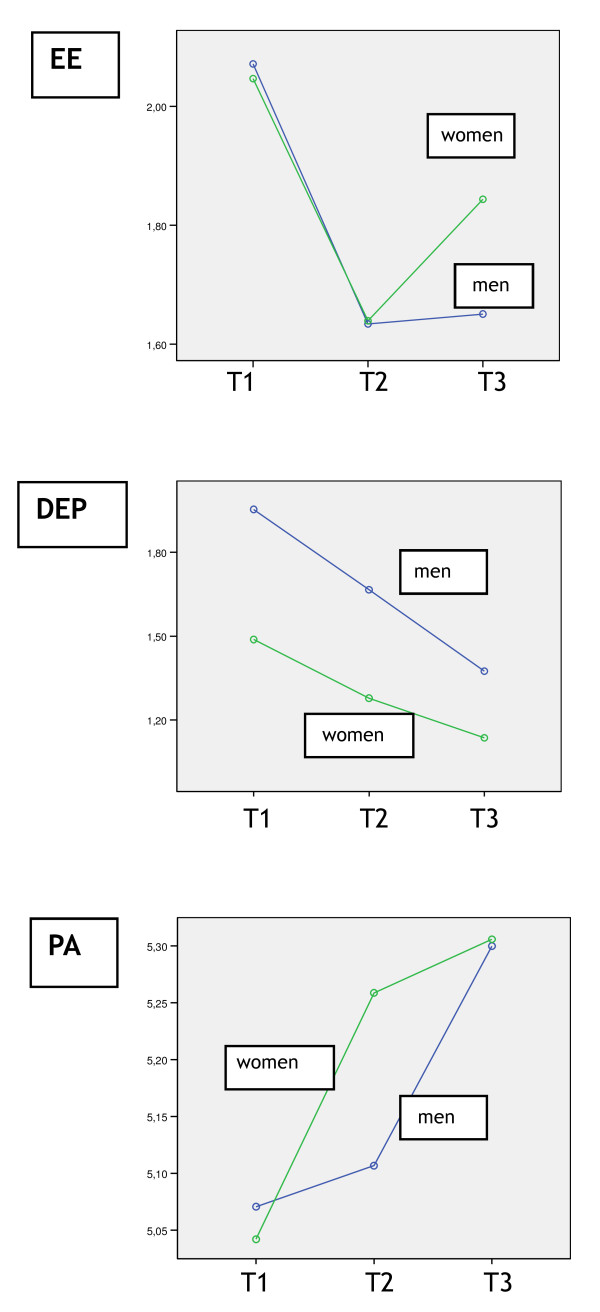
**Development of the three burnout dimensions over time**.

Table 2, as well as Figures 3 and 4 show the results of the preliminary analyses and the GLM repeated measures. Table 2 and Figure 3 show that in the first time lag - between 2002 and 2004 - the development of burnout was positive: the percentage of clinically burned out GPs decreased in this episode, from 20% to approximately 9%. In the second time lag, however, this percentage increased again, although the 2002 levels were not reached. The latter result was found in the panel group as a whole, as well as for men and women separately. More specifically, in the first time lag the *mean scores *for emotional exhaustion and depersonalization decreased and the *mean scores *for personal accomplishment increased. In the second time lag, particularly the level of emotional exhaustion increased again, while depersonalization and personal accomplishment developed in a more positive way. GLM 4 (repeated measures) indicates that these developments over time are significant for all three burnout dimensions (see Figure 4). In addition, we found a main effect of sex on depersonalization, implying that the level of depersonalization differs between men and women on *all three *time points. More specifically, male GPs report significantly *higher *levels of depersonalization, than female GPs. We found no main effects of sex on emotional exhaustion or personal accomplishment. Figure 4 indicates that for emotional exhaustion (particularly between T2 and T3) and personal accomplishment, the *development of mean scores over time *seems to differ between men and women. These differences were not significant though, that is, we could not find a significant interaction effect of time and sex (*p*-levels between .05 and .10).

**Table 2:** Development of burnout over time: means, standard deviations, and GLM repeated measures

		*Total* *(N = 212)*	*Main effect and effect size time*		*Men* *(N = 104)*	*Women* *(N = 108)*	*Main effect and effect size gender*		Interaction time*gender	
		***M (SD)***	***F (df)***	***p***	***M (SD)***	***M (SD)***	***F (df)***	***p***	***F (df)***	***p***

EE	T1	2.06 (1.09)	30.76 (2, 205)	.00	2.07 (1.25)	2.05 (0.93)	.20 (1)	.66	1.80 (2, 205)	.17
	T2	1.63 (0.98)	r_T1-T2 _= .48		1.63 (1.07)	1.63 (0.88)				
	T3	1.74 (1.05)	r_T2-T3 _= .14		1.65 (1.11)	1.83 (0.99)				

DEP	T1	1.72 (1.09)	27.15 (2, 205)	.00	1.95 (1.24)	1.50 (0.86)	10.31 (1)	.00	1.72 (2, 205)	.18
	T2	1.47 (0.97)	r_T1-T2 _= .27		1.66 (1.05)	1.29 (0.84)				
	T3	1.25 (0.86)	r_T2-T3 _= .25		1.37 (0.92)	1.13 (0.78)	r_m-f _= .22			

PA	T1	5.05 (0.74)	15.92 (2, 204)	.00	5.07 (0.73)	5.03 (0.75)	.26 (1)	.61	1.97 (2, 204)	.14
	T2	5.17 (0.78)	r_T1-T2 _= .18		5.11 (0.87)	5.24 (0.67)				
	T3	5.30 (0.64)	r_T2-T3 _= .19		5.29 (0.65)	5.29 (0.63)				

*Note*. EE = Emotional Exhaustion, DEP = Depersonalization, PA = Reduced Personal Accomplishment, T1 = 2002, T2 = 2004, T3 = 2006, only effect sizes for contrasts of significant main effects are reported.

### Research question 2: causal order of the three burnout dimensions among male and female GPs

First, we computed the bivariate Pearson correlations for all study variables (see Table 3). Table 3 shows that both the within wave and the across wave correlations among the three burnout dimensions are quite high for both sexes, although the relationship between emotional exhaustion and depersonalization is considerably stronger than the correlation between emotional exhaustion and personal accomplishment, particularly among men. Moreover, for women the across wave correlations between emotional exhaustion (Tn) and depersonalization in the next wave (Tn+1), is considerably higher than the correlation between depersonalization (Tn) and emotional exhaustion in the next wave (Tn+1). For men it is the other way around (time 1 - time 2) or less clear (time 2 - time 3). With regard to the relationship between depersonalization and personal accomplishment, it can be noticed that this correlation is weaker than the correlation between emotional exhaustion and depersonalization, for both sexes and within/across all waves.

**Table 3:** Zero-order Pearson correlations for males (N = 103, left-lower corner) and females (N = 207, right-upper corner)

*Variables*	*1*	*2*	*3*	*4*	*5*	*6*	*7*	*8*	*9*
1. EE (1)	--	.52*	-.11	.71*	.56*	-.26*	.54*	.34*	-.25*
2. DEP (1)	.76*	--	-.17	.28*	.65*	-.20*	.20*	.53*	-.14
3. PB (1)	-.26*	-.24*	--	-.09	-.05	.61*	.12	-.02	.62*
4. EE (2)	.74*	.59*	.33*	--	.60*	-.41*	.67*	.37*	-.33*
5. DEP (2)	.52*	.62*	-.29*	.69*	--	-.29*	.34*	.57*	-.20*
6. PB (2)	-.22*	-.20*	.53*	-.31*	-.36*	--	-.34*	-.17	.68*
7. EE (3)	.71*	.60*	-.20*	.71*	.49*	-.17	--	.46*	-.40*
8. DEP (3)	.56*	.60*	-.26*	.56*	.57*	-.21*	.75*	--	-.26*
9. PB (3)	-.19*	-.17	.56*	-.26*	-.26*	.60*	-.24*	-.29*	--

*Note*. Missing values were handled by listwise deletion. (1) = Time 1, (2) = Time 2, (3) = Time 3.

* *p *< .05.

In order to find out more about the causal direction of the relationships among the three burnout dimensions, we performed cross-lagged panel analyses.

**Figure 5:**
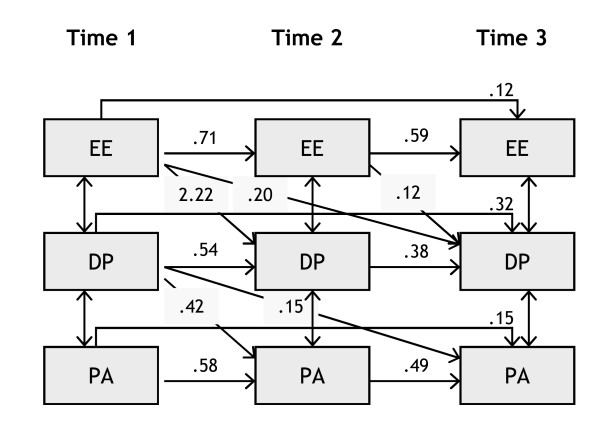
**Cross-lagged panel model for women**. Only significant cross-lagged effects are shown, within waves effects are not shown.

**Figure 6:**
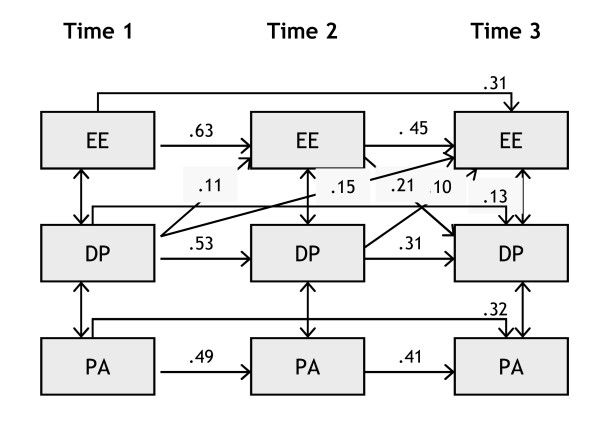
**Cross-lagged panel model for men**. Only significant cross-lagged effects are shown, within waves effects are not shown.

Table 4 and Figures 5 and 6 show the results of the cross-lagged panel analyses, using structural equation modelling. For both men and women, we compared the following models by means of the chi-square difference test and the AIC:

**Table 4:** Fit measures and chi-square difference tests of nested structural equation models for women and men

	*Χ^2^*	*df*	***comp***.	*ΔΧ^2^*	*Δdf*	*RMSEA*	*NNFI*	*CFI*	*AIC*	*SRMR*
Women (N = 104)										

M0	35.73*	18				.100	.94	.97	90.43	.074
**M1**	14.71	12	M0 - M1	21.02*	6	.048	.99	1.00	80.78	.053
M2	17.89	12	M0 - M2	17.84*	6	.066	.97	.99	83.18	.047
M3	10.54	9	M0 - M3	25.19*	9	.036	.99	1.00	82.14	.041
			M1 - M3	4.14	3					
			M2 - M3	7.35	3					

Men (N = 103)										

M0	21.68	18				.045	.99	1.00	75.56	.055
M1	13.82	12	M0 - M1	7.86	6	.027	.99	1.00	78.85	.043
M2	10.62	12	M0 - M2	11.06	6	.000	1.01	1.00	76.40	.044
M3	8.70	9	M0 - M3	12.98	9	.000	1.00	1.00	80.41	.042
			M1 - M3	5.12	3					
			M2 - M3	1.92	3					
**M3 adj**.	10.28	13	M0 - M3 adj.	11.40*	5	.000	1.01	1.00	74.10	.044

* *p *≤ .05.

*M0: *a model with only stability effects for all three burnout dimensions (T1 - T2, T2 - T3, and T1 - T3) and within wave associations between emotional exhaustion and depersonalization, and between depersonalization and personal accomplishment (in the form of correlated error terms for the endogenous variables and correlations for the exogenous variables, thus no direction was specified for these associations).

*M1: *similar to M0, but additionally including the cross-lagged relations between Tn emotional exhaustion to Tn+1 and Tn+2 (i.e., when applicable) depersonalization, and between Tn depersonalization and Tn+1 and Tn+2 personal accomplishment.

*M2: *similar to M0, but additionally including the cross-lagged relations between Tn depersonalization and Tn+1 and Tn+2 emotional exhaustion, and between Tn depersonalization and Tn+1 and Tn+2 personal accomplishment.

*M3: *a combination of M1 and M2.

It can be concluded from Table 4 that for women - according to the chi-square difference test - M1 is the best model (i.e., M1 is better than M0, *and *M3 is *not *better than M1 and M2). In addition, AIC of M1 is lowest. The practical fit indices are also very good for M1. This implies that for women the causal relationship between emotional exhaustion and depersonalization at a later moment predominates. For male GPs, it seems at first that none of the models including cross-lagged relationships is better than M0. The practical fit indices though, indicate that both M2 and M3 are the better models. Therefore, we omitted the non-significant effects in M3, and it appeared that this adjusted M3 is better than M0 (chi-square difference is significant, AIC of the adjusted M3 is lowest). This implies that for men the causal relationship between depersonalization and emotional exhaustion (with depersonalization being the predominant factor) is very important.

Figures 5 (for women) and 6 (for men) present the main findings graphically (omitting non-significant effects and the within wave effects). With regard to stability effects we can see that, for all burnout dimensions, the stability between T1 and T2 is highest, it is lower between T2 and T3, and even lower between T1 and T3.

With regard to the cross-lagged effects, it can be noticed that for women, the short term (T1 => T2; T2 => T3) and long term (T1 => T3) relationships between emotional exhaustion and depersonalization are quite clear, while the reverse relationships are not significant. In addition, there are significant cross-lagged relationships between depersonalization and reduced personal accomplishment. It seems that for women the development of burnout is generally in line with the Leiter and Maslach model, with emotional exhaustion being the core dimension of burnout, which triggers the development of depersonalization, and subsequently reduced personal accomplishment (through depersonalization).

For men (see Figure 6), we found significant short term (T1 => T2; T2 => T3) and long term (T1 => T3) relationships between depersonalization and emotional exhaustion, but not in the reverse direction. Only between T2 and T3, we found an additional relationship between emotional exhaustion and depersonalization. We did not find any significant cross lagged relationship between depersonalization and reduced personal accomplishment, nor modification indices suggesting direct relationships between emotional exhaustion and reduced personal accomplishment. This pattern of relationships, with depersonalization acting as a trigger of emotional exhaustion which may in turn further influence depersonalization, and reduced personal accomplishment developing independently from the other two burnout dimensions, is not in line with any of the theoretical models we presented earlier.

## Discussion and conclusions

The main purpose of the present study was to find evidence for possible differences in the prevalence and process of burnout between male and female GPs. By means of a three-wave panel study among a sample of Dutch GPs, we tested several theoretical burnout models and two possible sequences of burnout among male and female GPs separately.

### Change in prevalence of burnout over time (research question 1)

Generally, we observed an improvement in the burnout status between 2002 (T1) and 2004 (T2), while between 2004 and 2006 (T3) the situation deteriorated again. These developments might partly be explained by several changes in the working life of GPs. Since approximately 2000, the out-of-hours primary care provision is more and more organized in large-scale GP cooperatives, with generally 40 to120 GPs taking care of populations ranging from 50,000 to 500,000 inhabitants [43]. The GPs are assisted by doctor's assistants who perform telephone triage to prioritize treatment. Between 2002 and 2004 this development was at its peak. Research [8,43] shows that this development has led to a decrease in the average number of hours a GP is on call during a week (from 19 to 4). Moreover GPs experienced a reduced workload and fewer problems in the separation of work and private life. We believe that this development could have had it's main effect between 2002 and 2004 and may explain the decrease in burnout between T1 and T2. The increase in burnout between 2004 and 2006 could be explained by the fading out of the positive effect of the GP cooperatives or by the implementation of a new Health Insurance Act ("Zorgverzekeringswet") on January 1, 2006, which already cast its shadow before 2006. This act had large (administrative and financial) consequences for GPs who had to reorganize their administrative systems and working methods.

We did not find significant *sex *differences in the *change *in burnout prevalence over time, although women seem to develop more emotional exhaustion over time, despite the fact that they worked fewer hours than the male GPs in our sample. In addition, men scored - on average - higher on depersonalization.

### Process of burnout: causal order of the three dimensions of burnout among male and female GPs (research question 2)

As regards the developmental process of burnout, we found evidence for the fact that the aetiological process of burnout, that is the causal order of the three burnout dimensions, differs between male and female GPs. For *men*, *depersonalization *seemed to be the onset of burnout (note that men also had higher mean scores on depersonalization than women). Another salient finding among the male subgroup was that personal accomplishment seems to develop independently from the other two dimensions. Over time, we see that the feelings of personal accomplishment among male GPs keep increasing, despite feelings of depersonalization and emotional exhaustion. This finding suggests that for men reduced personal accomplishment is not a dimension of burnout. They appear to have a stable sense of self-efficacy that is not affected by exhaustion or cynicism.

For *women*, we found a strong influence of emotional exhaustion on depersonalization and of depersonalization on reduced personal accomplishment. In other words, for women burnout seems to be triggered by *emotional exhaustion*. We also found indications for a higher level of emotional exhaustion among women, particularly at time 3. This central role of emotional exhaustion for women is in line with, for instance, the Leiter and Maslach model. In addition, among women, feelings of personal accomplishment are affected by the other two burnout dimensions as well. Women who are exhausted and depersonalizing their patients, may start feeling guilty and less certain about their work and the quality of care they can provide.

These sex differences in the process of burnout are intriguing and may have large theoretical and practical implications. Below we describe several explanations for these sex differences in the developmental process of burnout.

First, the GPs' circumstances may have led to several gendered processes. The abovementioned introduction of the Health Insurance Act, for instance, along with all the hectic around it, may among men have lead to attracting management tasks and delegating caring and patient related tasks, while women may have tried to hold on to taking care of the patient at any cost; they did not depersonalize, but became exhausted. This feminine tendency to interact and communicate with patients on a high quality level may have been hampered, and this in turn led to exhaustion.

Second, women may have or perceive other (working) conditions and other individual characteristics than men, which in turn leads to a different development of burnout, with emotional exhaustion being more salient for women. Several studies [44,45] have indicated for instance, that women report more negative interaction between work and family life and that this interaction affected the development of emotional exhaustion only among women [45]. The same study [45] indicated that the individual factor "goal orientation", which was slightly more prevalent among male physicians, has a preventive effect for emotional exhaustion for men, but not for women. Goal orientation was defined as the active orientation towards long-term goals coupled with a reliance on one's own actions and feelings of personal responsibility to obtain these goals.

Finally, as mentioned above, men have a tendency to select avoidance coping strategies [29]. The construct depersonalization may also be considered an avoiding coping strategy, which may easily be adopted by men as a method to deal with a stressful situation. Our findings indicated that for men depersonalization leads to emotional exhaustion. This underlines the idea that psychological withdrawal can be an ineffective and dysfunctional coping strategy, which may affect the well-being, behaviour and performance of an employee. Moreover, for overly detached - male - GPs it might be more difficult to create a relationship with a patient, which may in the end influence the provision of care. This "indifferent" behaviour may cause patients to become more and more demanding, and at the same time cause feelings of overload within the GPs, which in turn triggers emotional exhaustion, and then the burnout process has started. At the same time, however, it should be noted that a certain level of detachment (i.e., *detached concern*) can be a very useful strategy for the prevention of ill-health and malfunctioning. In this light, one might argue that for both male and female GPs in our sample, a certain level of depersonalization might be a protective factor against burnout.

### Methodological reflections

Several methodological reflections should be made with regard to the present study. First, in the present study we aimed to find evidence for causality of relationships. However, in observational research, even if it is longitudinal and includes three waves, causality cannot be *proven*. Randomized controlled experiments, which are impossible and unethical to conduct in this research area, are needed for this purpose. In field studies, only causal inferences can be drawn [46].

In the second place, we measured the study variables at three fixed time points, with two year time lags while the processes we observed are continuous. Hence, there may have been a misfit between the actual causal lags among the burnout dimensions and the time lags used in the study. Moreover, the true time lags for the three burnout dimensions may differ from each other as well. In a study that uses a time lag of two years, a true three-months time-lag, might be better represented by the synchronous effects than by the lagged effects [32]. This time-lag problem is very difficult to solve and is intrinsic to longitudinal research in general. As theories rarely specify the correct causal lags, the causal processes may be captured best using different time lags in a study. Nevertheless, a misfit between the time lag used in the study and the actual causal lag, results in an underestimation of the true causal effects [32].

Third, there is the problem of attrition (i.e., panel mortality) that hampers CLPD. We used listwise deletion to arrive at the panel groups (complete data) for our cross-lagged panel analysis, and the panel groups in our study consisted of about 30% of the initial sample. In addition, our non-response analyses showed that the dropout among men was slightly higher than among women. This may have biased the results because of selective responses. The panel group did not differ from the dropouts with regard to the levels of burnout, though. Therefore, we concluded that no serious selection problems occurred. This type of missing data is called stratified MCAR and this could justify the use of listwise deletion as a method to handle these missing data when sex is taken into account in the analyses, as is the case in our study [42]. An issue related to the latter is the fact that in our study sample female GPs were overrepresented in comparison with the total population. Because we analyzed men and women separately we did not apply survey weighting. We made the decision to use a disproportionate sample because we aimed to establish sufficient variation in exposure to the model variables among both men and women. Moreover, female GP trainees account for 70% of the total group of GP trainees, so this study may also shed some light on future cohorts.

Finally, it should be noted that in the present study we ignored contextual and personal influences on the development of burnout. Hence, we did not control for the possible confounding effects of, for instance, work and personal characteristics, and gender characteristics (i.e., masculinity and femininity).

### Further research and practical implications

In spite of the reflections discussed above, we believe that the results of this study have important theoretical and practical implications, and give some directions for future research. First of all, the results implicate that in the current GP population there is no one valid burnout model for both men and women. This finding has implications for both the theorizing in the field on burnout, but also for preventive interventions with regard to burnout. These findings should be replicated in more longitudinal studies in other occupational groups. Moreover, the results of this study imply that in future burnout research it is advisable to consider both sex and gender differences, and maybe cultural differences as well. The present study particularly focused at sex differences between men and women. In future research though, it might be interesting to include the concept of gender identity (i.e., masculinity and femininity) [26]. Gender characteristics can vary considerably between individuals of the same sex, and therefore, sex *and *gender differences ought to be taken into account in future research regarding burnout [26]. It is interesting to focus on gender dynamics among physicians because the proportion of female physicians is increasing so rapidly and because we learned from previous research that professional identity and gender identity are profoundly influenced by the professional socialization in a hierarchical masculine culture in medical school and medical work.

With regard to practical implications, the results of this study suggest that in the first place it seems wise to pay attention to the mental health of general practitioners, and to take gender differences into account when doing so. For men, it might be wise to focus at coping capacities, in order to avoid withdrawal and isolation. For women (for whom emotional exhaustion is the onset of the burnout syndrome) it might be more useful to focus at the specific determinants of this fatigue-related dimension, and on learning how to prevent exhaustion by boundary-setting. In addition, Maslach and Leiter [6] suggest to detect burnout at an early stage and prevent the full blown development of burnout. They suggest that people with either high levels of emotional exhaustion or depersonalization show an "early warning" pattern. This would mean that for instance in screening for early burnout, we should focus on emotional exhaustion for women, and on depersonalization for men. This study also shows a need for gender-tailored stress interventions, which is in line with recent findings [47] about men and women reacting differently to stress-reducing interventions, and with recommendations from the European Commission [48] which has recognized a still-existing, large gender gap in almost all aspects of work quality, reconciling private and professional life and health and safety at the workplace. In this light the EC recommends the implementation of innovative and flexible work and leave arrangements.

## Competing interests

The authors declare that they have no competing interests.

## Authors' contributions

All authors conceived of the study. IH performed the statistical analyses and drafted the manuscript. YW designed the original project and collected the data together with MT. All authors contributed to the interpretation of the data, helped drafting the manuscript, and all authors read and approved the final manuscript.

## Pre-publication history

The pre-publication history for this paper can be accessed here:

http://www.biomedcentral.com/1471-2458/11/240/prepub
